# Research Progress on Epoxy Resins in Cultural Heritage Conservation

**DOI:** 10.3390/polym17131747

**Published:** 2025-06-24

**Authors:** Zirui Tang, Xinyou Liu, Xinhao Feng

**Affiliations:** College of Furnishings and Industrial Design, Nanjing Forestry University, Nanjing 210037, China; tangzirui@njfu.edu.cn (Z.T.); fengxinhao@njfu.edu.cn (X.F.)

**Keywords:** epoxy resins, cultural relics, modification process, restoration and conservation

## Abstract

Epoxy resins have been extensively employed in cultural heritage conservation as both adhesive and reinforcement materials owing to their exceptional bonding strength, relatively low toxicity, and cost-effectiveness. This review initially outlines the fundamental material characteristics of epoxy resins and subsequently examines their contemporary applications in artifact restoration. Subsequently, it synthesizes the research advancements documented over the past two decades, with a focus on critical challenges associated with their application in cultural heritage preservation, including susceptibility to aging, inherent brittleness, and prolonged curing time. The corresponding modification strategies are systematically examined, including strategies for aging resistance enhancement, toughness improvement, and rapid-curing techniques. Finally, potential future directions for epoxy resin applications in conservation are critically evaluated. This review provides a comprehensive analysis of epoxy resins’ performance and modification methodologies, thereby offering valuable insights to guide future research on its application in cultural heritage conservation.

## 1. Material Characteristics of Epoxy Resins

Epoxy resins, as versatile thermosetting polymers, have evolved significantly since their inception in the 1930s [1]. While maintaining their fundamental role in conventional applications (coatings, adhesives, composites), recent advances have expanded their utility in cultural heritage conservation [2]. This transition demands rigorous material evaluation, where epoxy’s unique combination of mechanical robustness, chemical resistance, and reversible adhesion meets the stringent requirements of artifact preservation [3].

The global epoxy resin industry has progressed through continuous innovation, with modern production primarily utilizing bisphenol-A/epichlorohydrin chemistry by a polycondensation reaction [4]. Current manufacturing employs either a one-step process for cost efficiency or two-step processes for high-performance grades, with Asia accounting for 60% of global capacity [5].

Epoxy resins exhibit a unique combination of high mechanical strength, excellent chemical resistance, distinct spectroscopic signatures, and superior optical clarity. To systematically evaluate these characteristics for conservation applications, Table 1 summarizes the key property ranges from standardized tests and authoritative references, providing a quantitative basis for material selection in cultural heritage preservation.

## 2. Application of Epoxy Resins in Cultural Heritage Conservation

Cultural artifacts, as material manifestations of human social activities within dis-tinct historical contexts, preserve invaluable historical data and constitute essential evidence for contemporary historical scholarship and reconstruction efforts [15]. The selection of conservation materials must adhere to rigorous principles, including minimal intervention, reversibility, discernibility, and long-term stability, due to the irreplaceable nature of these artifacts. These principles ensure that the authenticity and significance of cultural heritage are preserved while allowing for necessary conservation measures to be taken [16,17].

### 2.1. Application of Epoxy Resins in Bronze Artifacts

Epoxy resins have become valuable tools in the conservation of bronze artifacts due to their unique properties. In the restoration of a bronze bowman from Jebba, Fagg encountered the challenge of reinforcing a distorted base [18]. After initial straightening, it was discovered that the base required additional support due to weakened metal flanges. To address this, lengths of brass bar were brazed together to fit inside the base. These were secured using a cold-setting epoxy resin, which allowed the reinforcement to be non-integral and removable, ensuring future conservation efforts could be conducted without compromising the original structure. One of the key advantages of this restoration method is its reversibility. The epoxy resin allows the brass bars to be non-integral to the structure, meaning they can be removed at a later date if required. This is crucial for future conservation efforts, as it ensures that the original artifact remains largely unaltered and can be restored or treated differently if necessary. However, the use of brass bars and epoxy resin may not be entirely invisible, potentially affecting the visual integrity of the artifact. Although epoxy resins are generally durable, they can degrade over time, especially if exposed to harsh environmental conditions such as high humidity or fluctuating temperatures. This could lead to a weakening of the bond between the brass bars and the base, necessitating further restoration in the future.

Ma developed an innovative epoxy resin casting methodology for the reconstruction of fragmentary bronze artifacts, employing a plaster mold replication technique based on intact artifact components. This was followed by precision casting of pre-pigmented 815 epoxy resin to ensure chromatic continuity with the original artifact surface [19]. The cured replica underwent meticulous surface contouring to replicate the original curvature profile, before final assembly using a two-component epoxy adhesive system. The innovative approach exhibited substantial advantages compared to conventional tin–lead alloy techniques, including simplified procedures, enhanced safety due to the elimination of hazardous metalworking processes and superior aesthetic outcomes achieved through color-matched materials. While epoxy resins are generally durable, their use can make the restoration less reversible. Removing or altering the epoxy resin without damaging the original artifact can be challenging, which may limit future conservation options.

Liu adopted Antec’s three-ton-grade epoxy resin as the primary bonding material for bronze artifact restoration in their study [20]. This methodology accomplished dual objectives: secure interfacial adhesion and effective structural reinforcement of the original artifacts by implementing comprehensive surface application on metal fragments. The treatment markedly improved the structural stability of damaged sections, guaranteeing long-term conservation performance for thin-walled bronze objects, while achieving successful morphological reintegration of missing areas. However, while epoxy resins are generally stable, their aging behavior over decades or centuries remains uncertain compared to traditional materials like metallurgical bonding. Yellowing or brittleness may develop over time.

Iannucci et al. synthesized polymer/silica hybrid nanocoatings and graphene oxide-modified epoxy coatings to investigate the potential of hybrid polymer systems in corrosion protection for metallic artifacts [21]. The polymer/silica hybrid system synergistically integrates the inherent toughness and tunable surface properties of organic polymers with the exceptional hardness, chemical inertness, and thermal stability characteristic of silica-based inorganic matrices [22]. In contrast to pristine polymer systems, this nanocomposite demonstrates significantly improved scratch resistance and oxygen barrier properties, without compromising optical transparency, rendering it particularly suitable for heritage metal conservation applications [23]. The graphene oxide-reinforced epoxy system exhibits comparable enhancement in barrier efficacy and mechanical hardness relative to silica-based hybrid coatings, while offering additional advantages in interfacial adhesion strength [24].

These resins are widely used for reinforcing and consolidating fragile bronze surfaces, filling gaps and cracks, and providing a protective barrier against environmental degradation. However, there are also limitations that need to be considered. The adhesive strength of epoxy resins to metal surfaces is relatively low, necessitating the use of various modifiers containing functional groups capable of forming complexes with the metal [25].

### 2.2. Application of Epoxy Resins in Ceramic Artifacts

Epoxy resins have become prevalent materials in ceramic artifact restoration due to their exceptional bonding strength and durability. DeMestre’s research project systematically investigated the optimal concentration of fumed silica in a two-part epoxy resin for long-term color stability, chemical structure, and strength [26]. The study involves mixing a two-part epoxy resin with six different amounts of fumed silica to determine the ideal concentration that enhances these properties. The experimental approached includes artificially aging both resin samples and porcelain tiles repaired with the resin to assess long-term performance. The study demonstrates that the addition of fumed silica to two-part epoxy resins enhances the long-term durability of the resin. Meanwhile, the addition of fumed silica helps to minimize color change over time, ensuring that the restored porcelain maintains its original appearance. This is essential for maintaining the visual integrity of the artifact. While the study does not explicitly address the reversibility of the epoxy resin and fumed silica infills, the use of such materials generally makes the restoration less reversible. Removing or altering the resin without damaging the original porcelain can be challenging, which may limit future conservation options.

Huang et al. pioneered the application of epoxy resins as innovative conservation materials for restoring a Ming Dynasty blue-and-white porcelain vase [27]. Following comprehensive comparative analysis of the physico-chemical properties between traditional gypsum and modern epoxy resins, the team developed an innovative powder-mixed epoxy resin adhesive system. The epoxy resin adhesive system offers excellent mechanical compatibility with the porcelain material. It provides strong adhesion and structural support without causing damage to the original ceramic surface. The system also allows for the vase to withstand handling and display without compromising its structural integrity. However, removing or altering the epoxy resin without damaging the original vase can be challenging, which may limit future conservation options. This is an important consideration for conservators who may need to revisit the restoration in the future.

Correia employed 3D-printing technology to restore a 16th–17th century archaeological Ming Dynasty porcelain dish from the Monastery of Santa Clara-a-Velha in Coimbra, Portugal [28]. Given the low mechanical strength of plaster powder-based materials, the printed pieces were post-processed through infiltration with a two-part epoxy infiltrant to enhance structural integrity. The use of a two-part epoxy infiltrant significantly enhanced the mechanical strength of the 3D-printed pieces. This method ensured that the restored sections are durable and can withstand handling and display without compromising structural integrity. The epoxy’s resistance to environmental factors such as moisture and temperature fluctuations further contributes to the long-term preservation of the artifact. One significant drawback is the eventual yellowing of the epoxy adhesives due to their aging process. Over time, the epoxy may discolor, which can detract from the visual appearance of the restored porcelain dish.

**Figure 1:**
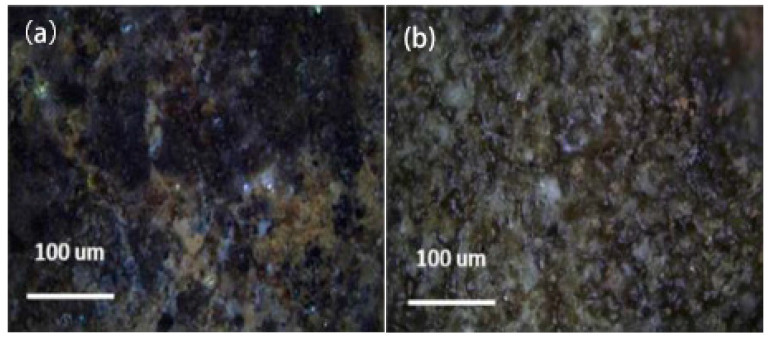
Epoxy adhesive surface and surface of the terracotta warriors: (**a**) Polarized micrograph of adhesive surface; (**b**) polarized micrograph of sample adhesive surface [29].

Tian characterized the interfacial morphology of epoxy-bonded terracotta warrior restoration surfaces [29]. Polarized light microscopy revealed ceramic particles adhered to the epoxy bonding interface, indicating sustained adhesive strength without significant degradation. This explained the observed ceramic material detachment from the substrate during debonding (Figure 1). A reversible composite adhesive was developed by combining epoxy with B-72. This formulation retains epoxy’s high bonding strength while enabling solvent-induced debonding. An optimal adhesive ratio was established that meets both terracotta restoration’s mechanical requirements and reversible conservation principles, providing key technical references for large-scale terracotta bonding systems. However, the presence of ceramic particles adhered to the epoxy bonding interface may affect the visual appearance of the restored surfaces, potentially requiring additional surface treatment to ensure seamless integration with the original artifact.

The materials’ primary advantages include their ability to form long-lasting adhesions that withstand environmental fluctuations while providing structural reinforcement to fragile ceramic pieces. However, several significant limitations must be considered. The irreversible nature of cured epoxy resins presents a fundamental conflict with contemporary conservation ethics that emphasize reversible treatments. Long-term stability issues, including yellowing and embrittlement, may compromise both the visual integrity and structural performance of restored artifacts. Material compatibility concerns arise from epoxy’s inherent rigidity, which can induce stress concentrations in thin-walled ceramics and potentially lead to new fractures.

### 2.3. Application of Epoxy Resins in Stone Artifacts

Epoxy resins are widely recognized as key materials in stone heritage conservation due to their superior mechanical and chemical properties. Huang et al. demonstrated that thorough mixing of epoxy resins with curing agents under low-temperature and ambient-pressure conditions yields highly stable curing performance, while maintaining a minimal shrinkage ratio [30]. The cured products exhibited exceptional electrical insulation properties, combined with superior physical and mechanical performance, underscoring their practical utility in cultural heritage preservation. However, the use of epoxy resins can make the restoration less reversible. Removing or altering the cured epoxy without damaging the original artifact can be challenging, which may limit future conservation options. This is an important consideration for conservators who may need to revisit the restoration in the future.

Kotik performed systematic impregnation experiments, immersing weathered stone specimens in epoxy resin–acetone solutions at varying concentrations [31]. The treatment significantly enhanced the compressive strength of the deteriorated stone substrates by 35–60%, demonstrating notable structural reinforcement. This study conclusively demonstrates the efficacy of epoxy resins in reinforcing weathered stone materials, offering both a scientific basis and a practical methodology for stone cultural heritage conservation. However, while less permanent than pure epoxy, the treatment still presents challenges for complete reversal after full curing.

**Figure 2:**
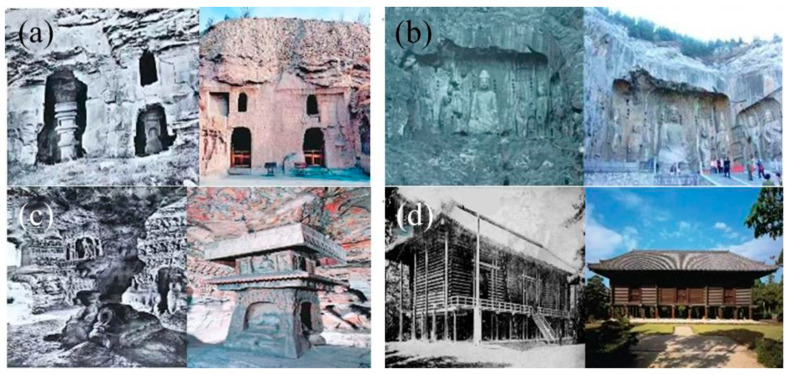
Comparison of the grottoes before and after restoration by epoxy resin [33]: (**a**) Yungang Grottoes; (**b**) the Great Shrine of Fengxian Temple; (**c**) polarized micrograph of sample adhesive surface; (**d**) Horyuji Temple.

Internationally renowned Chinese grottoes, including the Longmen Grottoes, Yungang Grottoes, Maijishan Grottoes, and Dazu Rock Carvings, have extensively utilized epoxy resins for structural consolidation. This practice has global counterparts, with Switzerland, Japan, the United States, and Italy widely applying epoxy resins in heritage conservation [32]. Notable applications include Japan’s successful restoration of Katsura Imperial Villa and Horyu-ji Temple; structural reinforcement of historic buildings in California and Louisville, Kentucky, USA; and experimental validation of specialized epoxy formulations in Andria, Italy [33]. Figure 2 provides comparative documentation of the grottoes before and after treatment, revealing significant pre-intervention deterioration (including weathering erosion and structural collapse) contrasted with nearly complete morphological restoration achieved through epoxy resin applications.

The mechanical compatibility of epoxy resins with stone materials ensures that restored artifacts can withstand handling and display conditions without compromising their structural integrity. However, the reversibility of epoxy resin treatments is limited, which may complicate future conservation efforts. Once applied, removing or altering the resin without damaging the original stone can be difficult, potentially limiting the options for future interventions. Despite these challenges, the overall advantages of epoxy resins in enhancing the structural stability and durability of stone artifacts make them a valuable tool in the field of cultural heritage conservation.

### 2.4. Application of Epoxy Resins in Wooden Artifacts

Epoxy resins have shown versatile applications in timber structure conservation, including reinforcing column capitals, reconnecting beam-purlin joints, and formulating epoxy-based repair fillers [34]. Epoxy resins are highly regarded in conservation practice due to their exceptional adhesive strength and optimal compatibility with lignocellulosic substrates. Documented applications encompass the structural consolidation of deteriorated timber elements at Shanxi’s Nanchan Temple (Xinzhou, China), alongside conservation interventions for significant architectural heritage in Brazil and Spain. These cross-cultural case studies systematically demonstrate the efficacy of epoxy resins in large-scale timber structural reinforcement. This material system facilitates straightforward application protocols while substantially improving load-bearing performance, maintaining both the structural authenticity and aesthetic integrity of historic buildings [35].

Fu et al. systematically evaluated six distinct adhesive formulations in a comprehensive screening study for lacquered wood artifact conservation [36]. The researchers meticulously analyzed the curing kinetics of these materials and performed accelerated thermal aging tests to obtain reliable data for long-term performance assessment. Experimental results revealed that the epoxy resin formulation demonstrated exceptional curing characteristics, achieving full polymerization within merely 5 min while retaining superior tensile strength. Nevertheless, given its comparatively reduced stability and durability under certain environmental conditions, prudent consideration of its limitations regarding aging resistance and thermal stability is essential for conservation applications (Table 2).

Bertolini et al. pioneered an innovative conservation methodology for structural timber elements salvaged from historic structures, incorporating selective removal of deteriorated wood portions followed by precision epoxy resin infill [37]. A rigorous comparative analysis between resin-treated specimens and untreated controls revealed that the epoxy system exhibited outstanding mechanical anchoring performance while preserving optimal substrate compatibility. Statistical validation of restoration outcomes demonstrated equivalent load-bearing capacity between intact timber members and those rehabilitated with epoxy-filled voids, conclusively establishing the material’s efficacy for structural reinforcement applications. However, epoxy repairs may require careful coloring and texturing to achieve seamless visual integration with aged wood surfaces, while structurally effective and differential thermal expansion between epoxy and wood requires monitoring to ensure lasting performance under cyclical environmental conditions.

**Figure 3:**
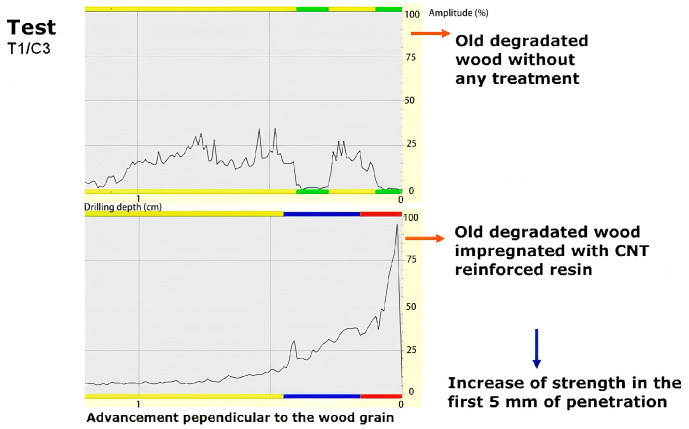
Comparison of densimeter test results before and after resin reinforcement with impregnated carbon nanotubes [38].

Cestari validated the effectiveness of carbon nanotube (CNT)-reinforced polymer resins in strengthening historic timber joints by conducting comparative pre- and post-impregnation analyses [38]. The tubular nanostructure of CNTs exhibited superior vapor permeability, markedly enhancing the moisture transfer capability of epoxy resins in wood treatments, thereby mitigating biological degradation via optimized moisture dispersion. Densitometric analysis revealed that the CNT-resin system attained a 5 mm penetration depth with significant strength improvements in impregnated zones, while indicating potential depth variations dependent on the wood’s initial degradation state and resin application dosage (Figure 3). However, while promising, the long-term behavior of CNT-resin composites in historic timber needs further study, particularly under cyclic humidity and temperature changes, and fully reversing the treatment without damaging the original wood fibers remains challenging, as with traditional epoxies.

Epoxy resins have emerged as highly effective materials for reinforcing historic timber structures, offering exceptional mechanical strength and compatibility with wood substrates. When properly applied, they can restore load-bearing capacity while preserving original material integrity. However, challenges remain regarding long-term stability under environmental cycling, aesthetic integration with aged wood surfaces, and complete reversibility. While epoxy treatments are particularly valuable for structurally compromised elements, their application requires careful consideration of wood condition, resin formulation, and conservation objectives to balance structural reinforcement with heritage preservation principles.

## 3. Challenges of Epoxy Resins in Cultural Heritage Conservation

Epoxy resin adhesives have gained widespread adoption in artifact restoration practices due to their superior performance characteristics, as demonstrated in the various applications reported in Section 2. These adhesives are highly valued for their strong bonding capabilities, chemical versatility, and overall effectiveness in preserving cultural heritage. However, it is important to recognize that these materials also exhibit certain limitations that can impact their long-term performance. These limitations include inadequate resistance to photo- and thermal aging, comparatively low impact strength, and extended curing durations.

### 3.1. Poor Resistance to Photo–Thermal Aging

The long-term preservation of cultural relics in diverse environments demands adhesives with enhanced resistance to environmental degradation factors, especially thermal and photo-oxidative aging. Such degradation mechanisms not only impair the adhesive’s interfacial bonding strength but can also induce chromatic alterations in artifacts, thereby compromising their historical authenticity and aesthetic significance [39,40].

In cultural heritage conservation, epoxy resins are widely employed as structural adhesives due to their strong bonding properties and ease of application. However, long-term exposure to natural sunlight or simulated solar irradiation—such as that produced by xenon arc lamps in artificial aging experiments—often leads to significant material degradation. This is predominantly manifested as yellowing, darkening, and a gradual loss of transparency [41]. Unlike high-energy UV radiation used in industrial applications, the photodegradation of epoxy resins in heritage conservation contexts occurs under lower-energy UV-A and visible light, which still initiate photochemical and photo-oxidative reactions over prolonged periods. These processes lead to scission of chemical bonds, the formation of chromophoric oxidation products, and the accumulation of degradation by-products that absorb visible light, thereby contributing to pronounced discoloration [42]. Furthermore, aging epoxy resins are known to exhibit a substantial increase in stiffness and elastic modulus, ultimately becoming brittle and prone to cracking or delamination. Such changes compromise the mechanical integrity of the bonded interface and may endanger the long-term stability of restored artifacts if not properly accounted for during material selection.

### 3.2. Poor Impact Resistance

In cultural heritage conservation, the bonding strength of adhesives represents a critical performance parameter. Insufficient adhesive strength can initiate multiple conservation challenges. Primarily, suboptimal bonding compromises restoration integrity, leading to inadequate mechanical performance in treated artifacts [19]. This may manifest as crack propagation or interfacial delamination, adversely impacting both conservation outcomes and exhibition viability. Secondarily, the long-term stability of artifacts becomes jeopardized, as sustained exposure to substandard bonding conditions promotes progressive material degradation and potential structural failure under mechanical loading. Additionally, the aesthetic integrity of artifacts may be compromised through visible defects arising from poor adhesive performance, ultimately diminishing their artistic value and visual coherence.

### 3.3. Slow Curing Speed

The utilization of epoxy resin adhesives in the restoration of delicate cultural heritage artifacts presents several technical constraints. The characteristically extended curing duration not only demands exceptional patience from conservators but also introduces significant handling challenges, particularly in achieving flawless interfacial bonding between fragments. Moreover, inherent procedural variations during adhesive application or polymerization phases may adversely affect restoration accuracy, potentially precluding optimal fragment realignment and seamless integration.

The mixture of epoxy resin and hardener undergoes an initial workable phase characterized by low viscosity and optimal spread ability at ambient temperature. As the cross-linking reaction progresses, the system exhibits an exothermic transition through gelation, evolving from a viscoelastic gel to a rigid elastomeric state. These curing kinetics directly influence fragment stability such that post-assembly dimensional drift manifests as progressive misalignment [20]. Such curing-induced displacements compromise restoration fidelity, ultimately resulting in perceptible interfacial discontinuities that reveal conservation interventions.

Furthermore, these adhesive systems demonstrate constrained pot life post-preparation, leading to diminished bonding performance. The workable period is governed by multiple interdependent parameters: resin-hardener chemistry, stoichiometric ratio, ambient temperature, and catalytic additives [41,42,43]. When the adhesive exceeds its optimal application window, storage-induced polymerization progresses to an extent that critically compromises its essential adhesive functionality through reduced wettability, impaired penetration into substrate micropores, and insufficient interfacial bonding strength development.

## 4. Research on Modification of Epoxy Resins

Epoxy resin adhesives are highly valued for their chemical versatility and strong adhesion, which are crucial for the restoration of both wooden and non-wooden materials. However, these adhesives also face critical challenges that impact their long-term performance. Addressing these limitations through performance modification is essential to maximize the efficacy of epoxy resins in cultural heritage restoration, improving the aging resistance, toughness, and rapid curing properties of epoxy resins.

### 4.1. Aging Resistance Modification of Epoxy Resins

It is essential to systematically investigate the fundamental causes of aging and elucidate the underlying mechanisms to scientifically mitigate the aging rate of epoxy resin materials. The most widely adopted strategies in both academic and industrial settings include the incorporation of organic small molecules for stabilization, the introduction of inorganic nanoparticles to improve material performance, and copolymerization and polymer blending modification techniques [44].

#### 4.1.1. Organic Small Molecule Stabilization Research

Organic small-molecule stabilizers can be categorized into three major types according to their functional mechanisms: antioxidants, light stabilizers, and thermal stabilizers. Antioxidants (e.g., phenolic compounds, amines, and thiodipropionate esters) primarily function through two mechanisms: scavenging free radicals generated during auto-oxidation, and decomposing hydroperoxides, thereby terminating chain propagation reactions in material aging processes [45]. Light stabilizers, particularly phenyl salicylate and hindered amine light stabilizers (HALS), function by either absorbing or screening ultraviolet radiation, thereby effectively suppressing photo-oxidation and subsequent material degradation. Thermal stabilizers (e.g., phosphites and polyols) predominantly protect materials from thermal degradation through chain-breaking donor action [46].

**Figure 4:**
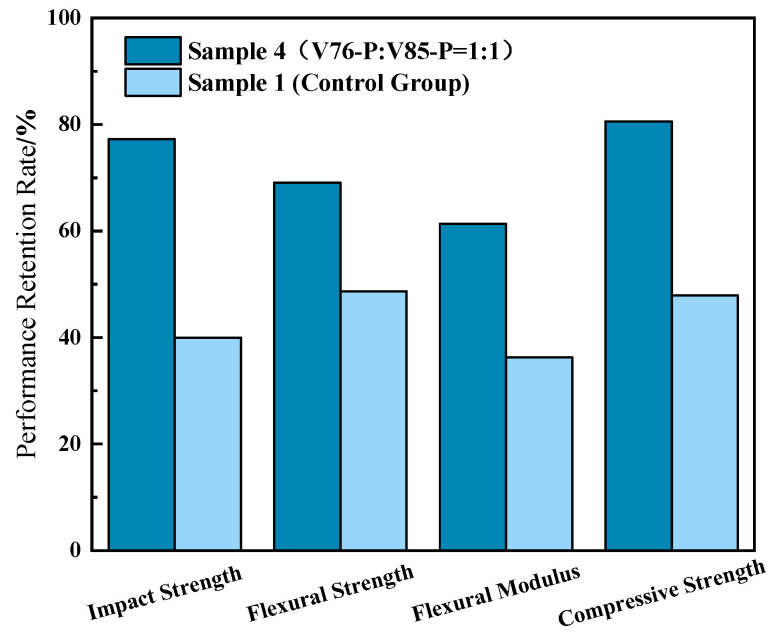
Retention rates of mechanical properties of recycled composites before and after anti-aging modification [47].

Cao et al. systematically investigated recycled composites comprising waste printed circuit board (PCB) powder and an epoxy resin, where composite antioxidants were incorporated to enhance material performance [47]. As depicted in Figure 4, the composite material subjected to accelerated thermal-oxidative aging at 100 °C for 100 days exhibited outstanding mechanical property retention exceeding 80%. These findings conclusively demonstrate the material’s exceptional thermal stability and superior durability under prolonged thermal-oxidative stress.

George examined the photostabilizing effects of different antioxidants on epoxy resin DEN438, identifying significant performance variations between Irganox1010 and Irganox1076 [48]. The results indicated that although both antioxidants were evaluated, only Irganox1010 substantially improved the material’s photostability, as evidenced by its effective suppression of carbonyl group formation during photoaging. However, under intense UV radiation, the photoprotective efficiency of Irganox1010 showed significant degradation, which was attributed to the photochemical decomposition of antioxidant molecules under high-intensity UV exposure, resulting in gradual depletion of their radical-scavenging capacity. This study elucidates the mechanistic limitations of organic stabilizers under extreme photodegradation conditions.

#### 4.1.2. Inorganic Nanofiller Protection Research

Inorganic nanofillers exhibit distinctive multifunctional properties, demonstrating remarkable efficacy in mitigating thermal-oxidative, hygrothermal, and photo-oxidative degradation processes in epoxy resins. These nanofillers afford comparable protection to organic small-molecule stabilizers while simultaneously providing superior physical barrier properties. They substantially improve the composite’s aging resistance through the formation of well-dispersed physical barrier networks within the polymer matrix. This methodology constitutes a principal strategy for enhancing the long-term durability of epoxy-based composites [49].

Park et al. developed two novel nanocomposite systems based on diglycidyl ether of bisphenol A (DGEBA) matrix, DGEBA/Al_2_O_3_ and DGEBA/SiC, incorporating nano-sized alumina (Al_2_O_3_) and silicon carbide (SiC) as reinforcing fillers via melt-blending technique [50]. Thermogravimetric analysis demonstrated that both nano-Al_2_O_3_ and SiC fillers substantially improved the thermal stability of the epoxy matrix, with the decomposition temperature increasing by approximately 40–50 °C compared to the pristine resin.

Qiu et al. engineered an advanced ternary nanocomposite system consisting of carbon fiber reinforcement, amino-functionalized multi-walled carbon nanotubes (NH_2_-MWCNTs), and an epoxy resin matrix [51]. The developed nanocomposite exhibited significantly enhanced resistance to hygrothermal aging, thermal-oxidative degradation, and salt spray corrosion. Furthermore, the incorporation of amino-functionalized carbon nanotubes accelerated the crosslinking curing reaction of the epoxy matrix while effectively reducing internal porosity, thereby enhancing the overall performance of the composite material.

#### 4.1.3. Copolymerization or Blending Modification Research

Copolymerization and polymer blending constitute two strategic modification approaches for molecular structure optimization and environmental aging resistance enhancement in epoxy resins [52]. Copolymerization chemically incorporates epoxy monomers with complementary functional monomers through covalent bonding, enabling precise control over polymer architecture and thermomechanical properties. This covalent integration approach facilitates the development of novel macromolecular architectures with designed chain configurations. In contrast, polymer blending physically combines epoxy matrices with secondary polymers through non-covalent interactions, creating heterogeneous systems with synergistic property combinations. This technique maintains the chemical integrity of each component while enabling favorable phase morphology through controlled processing conditions. Both methodologies fundamentally address the intrinsic aging vulnerabilities of epoxies by synergistically combining desirable attributes from different material systems [53].

**Figure 5:**
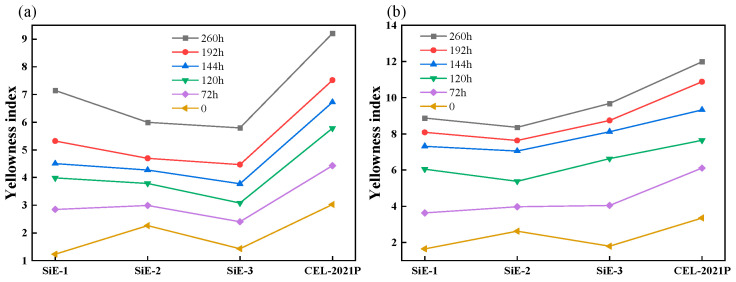
YI in (**a**) silicon ring resin and (**b**) CEL-2021P during thermal aging and UV [54].

Yang et al. synthesized two novel organosilane compounds, 2-(3,4-epoxycyclohexyl) ethyldimethoxysilane (EMDS) and dimethyldiethoxysilane (DMDES), with controlled stoichiometric ratios through an innovative co-hydrolysis and condensation reaction protocol [54]. The synthesized silane modifiers significantly improved the lap shear strength and fracture toughness of silicone resin composites, offering a versatile performance-enhancing platform for high-performance applications. Quantitative analysis revealed 40% lower UV-induced degradation rates compared to benchmark epoxy resin CEL-2021P, coupled with enhanced thermal stability and outstanding UV resistance (Figure 5).

Kim et al. systematically investigated the thermal stabilization mechanism of cyanate ester-modified bisphenol-A epoxy networks, employing a combination of spectroscopic, thermal, and rheological characterization techniques [55]. The research results demonstrated that increasing the cyanate ester content in the formulation led to simultaneous improvements in both the thermal stability and glass transition temperature (Tg) of the composite material. Consequently, these modifications effectively improve the overall thermal stability and thermal decomposition resistance of the composite system.

### 4.2. Toughening Modification of Epoxy Resins

The insufficient mechanical strength of epoxy resin adhesives may lead to crack formation or structural damage in restored cultural relics. Researchers have developed multiple strategies to enhance the resin matrix by incorporating fillers to address these limitations. These methods primarily employ highly elastic polymers (e.g., rubber or thermoplastic materials) and inorganic nanomaterials for composite modification, aiming to simultaneously improve the toughness and strength of epoxy resins [56,57,58,59].

#### 4.2.1. Rubber Elastomer Toughening

The incorporation of rubber-modified epoxy resins effectively reduces internal stress while substantially improving toughness. This modification occurs through chemical crosslinking between the rubber molecules’ active functional groups and the epoxy resins’ reactive groups (epoxy and hydroxyl groups), forming a block copolymer structure. The rubber phase promotes energy dissipation within the matrix, thereby effectively suppressing crack propagation [60,61].

**Figure 6:**
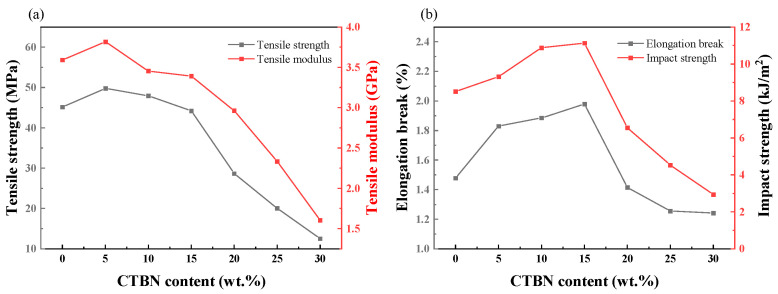
Mechanical properties of composites with different CTBN contents: (**a**) tensile strength and tensile modulus and (**b**) elongation at break and impact strength [62].

Bian et al. systematically evaluated the toughening effect of carboxyl-terminated liquid butadiene–acrylonitrile rubber (CTBN) as an epoxy resin modifier, with results illustrated in Figure 6. The uniformly dispersed CTBN phase in the epoxy matrix significantly improved the composite’s toughness at CTBN concentrations of 5–15%. The toughening mechanism arises from the CTBN island phase’s stress-distribution capability, which enhances fracture resistance, while the average CTBN particle size increases proportionally with concentration [62].

Dou et al. fabricated rubber-toughened epoxy resins with controlled phase-separated morphologies by adjusting the acrylonitrile content in amine-terminated butadiene–acrylonitrile rubber (ATBN) [63]. Their results demonstrated a clear dependence of rubber particle size on the ATBN’s acrylonitrile concentration. These findings offer critical insights for optimizing rubber-modified epoxy systems’ mechanical performance.

In an alternative strategy, Li et al. utilized polysulfide rubber as a toughening modifier for epoxy resins [64]. The distinctive thioether linkages in polysulfide rubber’s molecular structure significantly enhanced polymer chain mobility, providing an effective mechanism for epoxy resin toughening.

#### 4.2.2. Thermoplastic Resin Toughening

Thermoplastic resins demonstrate exceptional toughness and thermal stability, rendering them essential components in composite materials. When incorporated into epoxy systems, thermoplastic resins simultaneously enhance both toughness and other critical performance metrics while maintaining the modulus of the cured product [65,66]. This synergistic improvement ensures composite stability and reliability under multiaxial stress conditions.

**Figure 7:**
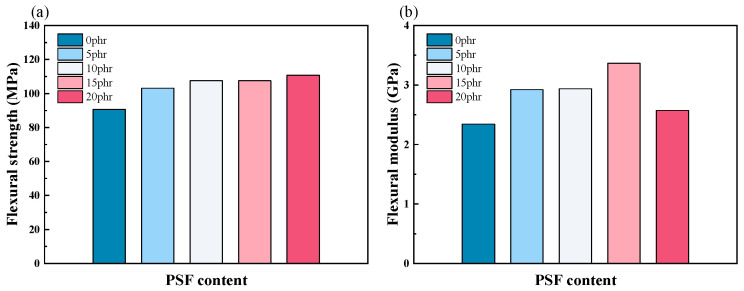
(**a**) The flexural strength and (**b**) modulus of PSF/epoxy blends as a function of PSF content [67].

Sun et al. utilized polysulfone (PSF) as a toughening agent for epoxy resin systems [67]. Their results revealed that while PSF modification preserved the epoxy’s curing mechanism, it demonstrated excellent matrix compatibility while accelerating the curing kinetics. The PSF-modified composites exhibited enhanced flexural strength and increased elastic modulus (Figure 7).

Zhang et al. synthesized hydroxyl-terminated polyurethane prepolymers through the reaction of polyethylene glycol with isophorone diisocyanate [68]. These prepolymers were then employed to modify epoxy resins, yielding substantial enhancements in multiple material properties. The modified epoxy system demonstrated concurrent improvements in elongation at break, tensile shear strength, impact strength, toughness, and adhesive performance at an optimal loading of 15 phr polyurethane prepolymer.

#### 4.2.3. Toughening with Inorganic Nanoparticles

A prevalent strategy for epoxy resin modification incorporates inorganic nanoparticles into the matrix via physical or chemical methods [69]. This approach substantially improves both mechanical and physical properties. In polymer science, nanoscale inorganic fillers have attracted significant attention owing to their exceptional property-enhancing capabilities. These nanofillers are widely employed in epoxy composites, enhancing mechanical properties (hardness, tensile strength, wear resistance) while simultaneously improving optical, thermal, and electrical properties. These multifunctional enhancements facilitate the customized design of epoxy composites for specific industrial applications [70].

**Figure 8:**
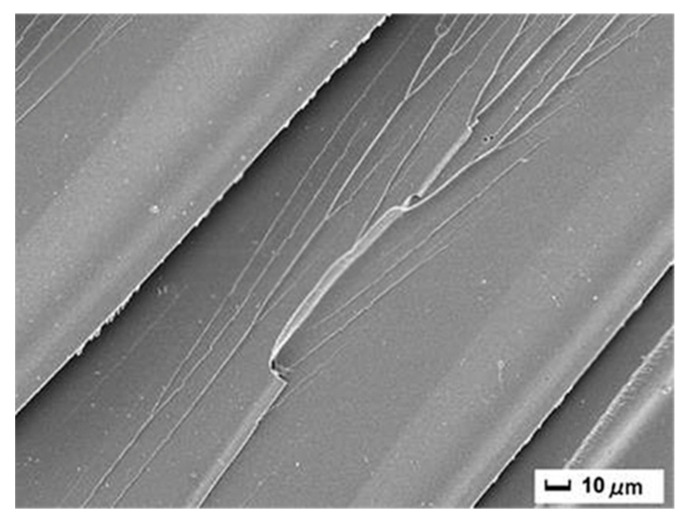
SEM images of the brittle fracture surface of pure EP [71].

**Figure 9:**
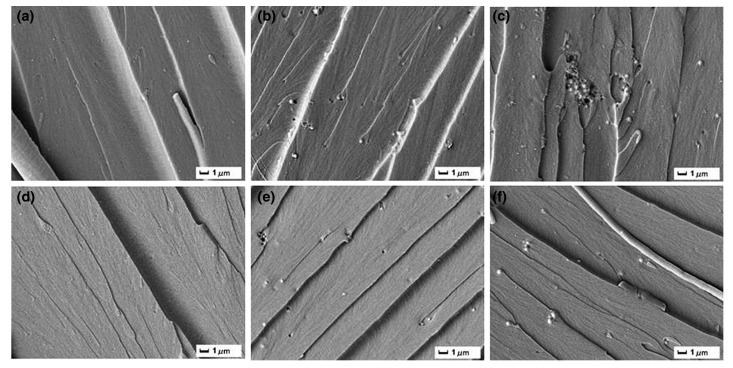
EM images of the brittle fracture surface of EP/SiO_2_ and SiO_2_-TETA composite materials containing (**a**) 100 nm, (**b**) 300 nm, and (**c**) 500 nm of SiO_2_, and (**d**) 100 nm, (**e**) 300 nm, and (**f**) 500 nm of SiO_2_-TETA [71].

Ai et al. prepared size-tunable silica nanoparticles through an aqueous sol–gel process, followed by triethylenetetramine functionalization to improve epoxy matrix toughness [71]. The results revealed a pronounced particle size dependence of toughening efficiency (Figure 8 and Figure 9), with 100 nm silica particles showing optimal performance. Their maximized specific surface area yielded the greatest enhancement in fracture resistance.

Mirsalehi et al. fabricated DGEBA-based epoxy nanocomposites containing multi-walled carbon nanotubes (MWCNTs) at different loading concentrations [72]. The incorporation of 1.0 wt% MWCNTs substantially improved the nanocomposite’s elastic modulus, hardness, resistance to plastic deformation, and wear characteristics.

**Figure 10:**
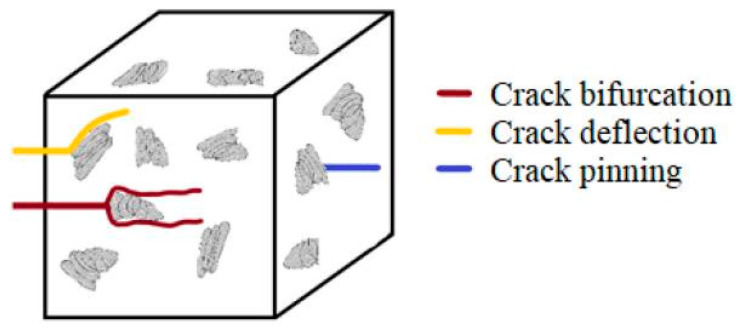
The schematic micro-scale toughening mechanisms of the nanoplatelets in epoxy-based nanocomposites [73].

Bahari-Sambran et al. examined the synergistic reinforcement effects of surface-functionalized montmorillonite (MMT) and graphene nanoplatelets (GNPs) in epoxy nanocomposites, analyzing both nanofiller dispersion and mechanical enhancement (Figure 10). The optimal nanocomposite containing 0.15 wt% GNPs and 1 wt% MMT showed 19% greater tensile strength and 17.4% higher flexural strength than the neat epoxy [73].

#### 4.2.4. Toughening with Hyperbranched Polymers

Hyperbranched polymers (HBPs) possess unique characteristics including low viscosity, high free volume, and excellent solubility, making them ideal toughening agents for epoxy resins owing to their numerous functional groups. The toughening mechanisms include cavitation-induced void formation, crack tip blunting, matrix shear yielding, and increased deformation capacity. Importantly, hydroxyl- or epoxy-terminated HBPs participate directly in the curing reaction, functioning as reactive modifiers that substantially enhance the composite’s mechanical properties [74].

Chen et al. synthesized a novel hyperbranched alkoxysilane (HAHBPs) via an A2 + B3 polycondensation of diethylene glycol with triethoxysilane under solvent-free, catalyst-free conditions [75]. HAHBPs increased flexural strength by 25.7% and impact strength by 148%, demonstrating exceptional reinforcement efficiency at 20 wt% loading in E51 epoxy resin. In a related study, Hu et al. prepared a hyperbranched polysiloxane by reacting vinyltriethoxysilane with diethylene glycol under nitrogen protection. This modifier improved elongation at break by 84.5%, KIC by 330.1%, and impact strength by 82.3% at 3 wt% loading, showing outstanding mechanical property enhancement [76].

**Figure 11:**
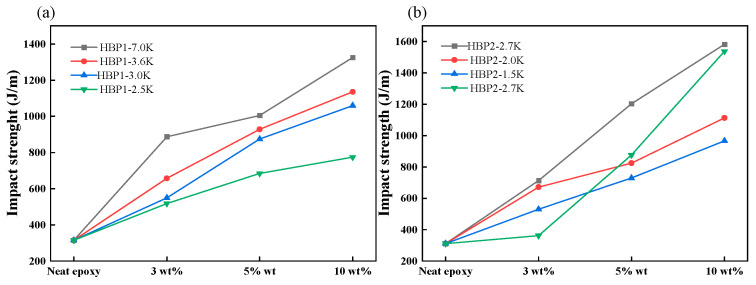
Impact strength of (**a**) epoxy/HBP1 and (**b**) epoxy/HBP2 blends with different molecular weights and HBP loadings [77].

Park et al. synthesized two molecular-weight-controlled hyperbranched poly (methyl acrylate) derivatives, poly (methyl acrylate diethanolamine) and poly (methyl acrylate ethanolamine), which were subsequently blended with epoxy resin and polyetheramine [77]. Incorporation of 10 wt% HBPS yielded a 270% enhancement in impact resistance (Figure 11), conclusively demonstrating hyperbranched polymers’ exceptional toughening capability in epoxy systems.

#### 4.2.5. Bio-Based Toughening

Amid growing energy demands and environmental concerns, developing sustainable bio-based approaches for epoxy resin modification has become a prominent research frontier. Researchers are investigating diverse renewable resources for sustainable epoxy toughening [78]. These bio-based materials reduce fossil fuel dependence while lowering production carbon emissions, advancing sustainable materials development.

Pham synthesized rice husk-derived nanosilica that enhanced fracture toughness by 16.3% at an ultralow loading of 0.07 phr [79]. Yang prepared nano-TiO_2_/cellulose (TiO_2_/RC) composites via microwave-assisted synthesis, achieving 38% higher tensile strength and 40% greater impact toughness at 10 phr loading [80]. Xu developed an economical carboxylated tannic acid (CATA) hyperbranched toughener for solvent-free epoxy systems [81]. Suganya et al. fabricated a biocomposite containing 30 vol% aloe vera fibers and 0.5 vol% pistachio shell particles, significantly improving flexural, tensile, and impact properties [82].

### 4.3. Rapid Curing Modification of Epoxy Resins

In the conservation of cultural heritage, especially in structural consolidation or gap filling, the use of rapidly curing epoxy resins presents both opportunities and critical challenges. While acceleration of curing reactions can offer practical advantages—such as reduced intervention time, minimized exposure to environmental fluctuations, and faster stabilization—such modifications may compromise long-term stability, compatibility with original materials, and reversibility, which are essential in conservation ethics.

Additives and curing agents used to enhance curing speed can significantly influence the formation of the crosslinked network, thus altering mechanical, thermal, and chemical properties [83]. In heritage applications, however, such enhancements must be balanced with considerations of aging behavior, visual impact, and potential retreatability. Therefore, rapid curing formulations require thorough evaluation under conservation-relevant conditions before being adopted.

#### 4.3.1. Amine-Based Accelerators in Epoxy Systems for Conservation Use

Amine-based curing agents are known for their high reactivity and are widely used in engineering applications. However, their fast reaction rates and associated heat release raise concerns in heritage materials, which may be sensitive to localized thermal or mechanical stresses.

In industrial studies, Yang et al. demonstrated that incorporating 1,3-cyclohexanedimethanamine and isophoronediamine into DQ204H epoxy systems significantly enhanced curing rates and mechanical properties [84]. However, these conditions (e.g., tensile strength up to 67.2 MPa and impact toughness of 52.95 kJ/m^2^) may not be directly applicable to fragile substrates such as ancient wood or stone. Similarly, Ren et al. showed that adding imidazoles to aromatic amine systems resulted in increased flexural and tensile strength [85], but the rapid gelation times (within 10 min) may impede careful application in conservation tasks where extended working time is necessary.

#### 4.3.2. Anhydride and Hybrid Curing Agents: A More Controlled Approach

Anhydride-based curing systems offer a slower reaction profile, which can be advantageous in conservation, allowing more controlled application and reduced exothermic stress. Yang et al. observed that dimethylbenzylamine acted as a catalyst in methyltetrahydrophthalic anhydride–epoxy systems, lowering the curing onset temperature and improving processing efficiency [86]. This kind of controlled acceleration could be more suitable for sensitive conservation environments.

Furthermore, Qiu et al. synthesized an EC-g-MPA anhydride curing agent, where a strong correlation was found between heating rate and cure rate [87], suggesting that thermal management can be used as a parameter to fine-tune performance. Liu et al. and Qiu et al. also explored hybrid systems using amine-modified fillers (such as aminated carbon nanotubes), which not only catalyzed curing but also enhanced mechanical bonding [88]. Despite their promise in structural composites, their application in cultural heritage remains speculative and requires specific long-term performance validation.

## 5. Conclusions and Perspectives

Epoxy resins have demonstrated significant utility in cultural heritage conservation for specific applications due to their superior adhesion, mechanical properties, and chemical resistance. However, their use is context-dependent, with regional variations in conservation practice. For instance, while still employed for metal and ceramic restoration in many regions, their application in the restoration of natural stones and built heritage has declined due to compatibility concerns. These properties enable diverse conservation applications: artifact repair through fragment reintegration and void filling to restore structural and aesthetic integrity; object consolidation by mechanically reinforcing fragile materials; protective surface coatings that resist environmental degradation; and replica fabrication for exhibition and study.

Despite their advantages, conventional epoxy formulations present several limitations necessitating further research and development. Key limitations include insufficient photostability and thermal aging resistance, limited impact strength, and sluggish curing kinetics, which may impair long-term artifact preservation and necessitate future re-treatment. To address these issues, researchers have developed three primary modification approaches: photothermal stabilization using organic stabilizers and nanofillers; toughness enhancement through elastomers, thermoplastics, nanoparticles, or hyperbranched polymers; and curing acceleration via reactive amine/anhydride systems.

To optimize the performance of epoxy resins in conservation practice, several key recommendations emerge from the current research. First, modification approaches should carefully balance bonding strength with reversible solubility in appropriate solvents to maintain both effective adhesion and the principle of reversibility in conservation treatments. Second, preference should be given to inorganic modification methods that better align with the aging characteristics of heritage materials. Third, comprehensive evaluation of newly developed epoxy formulations through long-term testing on simulated artifacts is essential before their application to genuine cultural objects.

Looking ahead, the future development of epoxy resins for heritage conservation should prioritize four key directions aligned with modern conservation principles: (1) performance optimization focusing on material compatibility, enhanced aging resistance, and color stability; (2) functional diversification incorporating reversible systems, antimicrobial properties, and controlled flame retardancy; (3) sustainable formulations reducing volatile organic compounds and health hazards; and (4) compliance with minimal intervention principles through tunable crosslinking density and solvent-assisted reversibility. As fundamental materials in modern conservation practice, epoxy resins will continue to play a pivotal role in heritage preservation, with ongoing technological advancements promising ever more sophisticated solutions to meet the complex challenges of safeguarding cultural heritage for future generations.

## Figures and Tables

**Table 1:** Major properties of epoxy resins.

Property Category	Specific Property	Typical Value Range	Test Method/Conditions	Reference
Mechanical Properties	Tensile Strength	45–85 MPa	ASTM D638	[6]
	Elastic Modulus	2.1–3.5 GPa	ISO 527	[7]
	Elongation at Break	3–8%	ASTM D638	[8]
Chemical Resistance	Acid Resistance (20% H_2_SO_4_)	<5% weight loss (24 h)	ASTM D543	[9]
	Alkali Resistance (10% NaOH)	<8% weight loss (24 h)	ASTM D543	[1]
	Solvent Resistance (acetone)	Swelling <15%	ASTM D471	[1]
Spectroscopic Properties	FTIR Characteristic Peaks	915 cm^−1^ (epoxy), 1505 cm^−1^ (aromatic)	FTIR spectroscopy	[10]
	NMR Chemical Shifts (^1^H)	δ 2.5–3.5 (epoxy CH_2_), δ 6.5–7.5 (aromatic)	^1^H NMR (400 MHz)	[11]
Optical Properties	Refractive Index	1.55–1.60 (cured)	ASTM D542	[12]
	Light Transmittance	85–92% (400–700 nm)	ASTM D1003	[13]
	Yellowing Index	<5 (initial), <15 (1000 h UV)	ASTM E313	[14]

**Table 2:** Comparison of adhesive performance test results [36].

Evaluation Metrics	Curing Time	Wood Tensile Strength	Fabric Peel Strength	Post-Aging Bond Strength Change
Fish Bladder Glue	12 h	Strong	Moderately Strong	Minor Change
Bone Glue	12 h	Moderately Strong	Moderately Strong	Stable
Shellac	12 h	Strong	Strong	Stable
Rice Paste	24 h	Weak	Strong	Minor Change

## Data Availability

The original contributions presented in this study are included in the article.
